# Supplementary material for: Record linkage studies of primary care utilisation after release from prison: A scoping review protocol
Source: PLoS One. 2023 Aug 25;18(8):e0289218. doi: 10.1371/journal.pone.0289218 (PMC10456167; doi:10.1371/journal.pone.0289218)
Supplement: S1 Appendix — (DOCX) [file pone.0289218.s002.docx]

**Supplementary material**

Record linkage studies of primary care utilisation after release from prison: a scoping review protocol

**Authors**

Janine A. Cooper ^1,2^, Siobhan Murphy ^1,2^, Richard Kirk ^3^, Dermot O’Reilly ^1,2^, Michael Donnelly ^1,2^

^1^ Centre for Public Health, Queen's University Belfast, Royal Hospitals Site, Grosvenor Road, Belfast, UK

^2^ Administrative Data Research Centre Northern Ireland (ADRC NI), Centre for Public Health, Queen's University Belfast, Royal Hospitals Site, Grosvenor Road, Belfast, UK

^3^ South Eastern Health and Social Care Trust, Ulster Hospital, Dundonald, UK

**S2 appendix**

**S2 appendix**

**Search strategy for MEDLINE**

Ovid MEDLINE(R) ALL <1946 to February 01, 2023>

1 General Practice/ or Family Practice/ 78099

2 General Practitioners/ 10256

3 GP.mp. 48693

4 Physicians, Family/ 17126

5 Primary Health Care/ 90209

6 primary care.mp. 142109

7 home visit*.mp. or House Calls/ 12539

8 Family Health/ 24126

9 Physicians, Family/ or Physicians, Primary Care/ 21337

10 Nurse Practitioners/ 18753

11 Community Pharmacy Services/ 5653

12 Community Dentistry/ 1233

13 Optometrists/ or Optometry/ 5823

14 Prisoners/ 18209

15 Prisons/ 11017

16 after release.mp. 4474

17 prison release.mp. 154

18 release* from prison.mp. 602

19 ex-prisoner*.mp. 214

20 recently released.mp. 2127

21 1 or 2 or 3 or 4 or 5 or 6 or 7 or 8 or 9 or 10 or 11 or 12 or 13 348163

22 14 or 15 or 16 or 17 or 18 or 19 or 20 30977

23 21 and 22 449

24 limit 23 to (english language and humans and yr="2012 -Current") 185
