# Supplementary material for: Record linkage studies of primary care utilisation after release from prison: A scoping review protocol
Source: PLoS One. 2023 Aug 25;18(8):e0289218. doi: 10.1371/journal.pone.0289218 (PMC10456167; doi:10.1371/journal.pone.0289218)
Supplement: S2 Appendix — (DOCX) [file pone.0289218.s003.docx]

**Supplementary material**

Record linkage studies of primary care utilisation after release from prison: a scoping review protocol

**Authors**

Janine A. Cooper ^1,2^, Siobhan Murphy ^1,2^, Richard Kirk ^3^, Dermot O’Reilly ^1,2^, Michael Donnelly ^1,2^

^1^ Centre for Public Health, Queen's University Belfast, Royal Hospitals Site, Grosvenor Road, Belfast, UK

^2^ Administrative Data Research Centre Northern Ireland (ADRC NI), Centre for Public Health, Queen's University Belfast, Royal Hospitals Site, Grosvenor Road, Belfast, UK

^3^ South Eastern Health and Social Care Trust, Ulster Hospital, Dundonald, UK

**S3 appendix**

**S3 appendix**

**Data charting form**

**General Information**

|  | **Information as stated in publication** |
| --- | --- |
| **Initials of researcher completing the data charting form** |  |
| **Date of completion of data charting form** |  |
| **First author (i.e. lead author on paper)** |  |
| **Year of publication** |  |
| **Study title** |  |
| **Journal name** |  |

**Methods**

|  | **Information as stated in publication** |
| --- | --- |
| **Study design** |  |
| **Sources of linked data** |  |
| **Custodial setting (e.g. prison, jail)** |  |
| **Primary care service(s) (e.g. general practice, pharmacy, dentist, optician)** |  |
| **Method of data linkage** |  |
| **Years of data linked** |  |
| **Time period examined after prison release** |  |
| **Source of comparator data** |  |
| **Matching** |  |

**Participants**

|  | **Information as stated in publication** |
| --- | --- |
| **Setting location (country)** |  |
| **Number of participants in cohort/number of cases** |  |
| **Number of controls/number in comparator group** |  |
| **Inclusion of participants** |  |
| **Exclusion of participants** |  |
| **Age (state time point e.g. release)** |  |
| **Gender** |  |
| **Race/ethnicity** |  |

**Outcomes**

|  | **Information as stated in publication** |
| --- | --- |
| **Primary and secondary outcomes (as reported)** |  |

**Statistical analysis**

|  | **Information as stated in publication** |
| --- | --- |
| **Statistical analysis (as reported)** |  |

**Results**

|  | **Information as stated in publication** |
| --- | --- |
| **Results (for outcomes reported)** |  |
